# Perception of Mothers about Their Daughters Future in Rural Karnataka

**DOI:** 10.4103/0970-0218.69284

**Published:** 2010-07

**Authors:** Rashmi

**Affiliations:** Department of Community Medicine, KS Hegde Medical Academy, Derlakatte, Mangalore-18, India. E-mail: csrashmi@yahoo.com

Sir,

India is predominantly a rural area and a land with ancient values. The growing concern is the decreasing male and female ratio and the need for program for the upliftment of women in the nation. But the daughter’s future is shaped by her first teacher that is mother. What do these mothers think about their daughter in future? So a study in the central Karnataka region (Davangere taluk) was conducted in order to understand the mothers’ perception and ambition about their daughters. All mothers with a girl child aged 5–15 years were the participants. The taluk was divided into 4 quadrants and 2 villages from each quadrant were randomly selected and 30 houses in each village by systematic random sampling were picked.

The results depicted that 41% of the mothers were wishing their daughters to study as long as they wished and 32% felt that 10^th^standard was maximum necessity. A total of 69% of the mothers wanted their girl child to be married at 20–25 years of age. Early pregnancy was told to be risky by 53% of the mothers. 54% of the mothers wanted daughters to be economically independent [Table 1].

**Table 1:** Mothers’ perception with age group, religion, and mothers’ education

Age group of mothers in (years)	Mother’s ambition about daughter’s education			Mother’s knowledge of early pregnancy risk	Mothers wishing daughter’s economical independence	Total
	10^th^ standard	Graduation/PG/professional	As long as she wishes			
20–30	14	30	42	54	60	86
	16.3%	34.88%	48.84%	62.79%	69.8%	36.44%
30–40	48	28	34	50	48	110
	43.6%	25.45%	30.9%	45.45%	43.6%	46.61%
40+	14	6	20	20	20	40
	35%	15%	50%	50%	50%	16.95%
Religion						
Hindu	46	38	66	88	88	150
	30.7%	25.3%	44%	58.7%	57.7%	63.6%
Muslim	30	26	30	36	40	86
	34.9%	30.2%	34.9%	41.9%	46.5%	36.4%
Education of mother						
Nil	40	20	34	42	48	66
	42.5%	21.2%	36.2%	44.7%	51.1%	27.9%
Up to 7^th^	24	12	30	34	34	32
	36.4%	18.1%	45.5%	51.5%	51.5%	13.6%
Up to 10^th^	6	8	18	18	22	44
	18.7%	25%	56.5%	56.3%	68.8%	18.6%
>10^th^	6	24	14	30	34	94
	13.6%	54.5%	31.8%	68.2%	77.3%	39.8%

Total	76	64	96	124	128	236
	32.2%	27.11%	68.64%	52.5%	54.2%	

Significance of age of mother		*χ*^2^=20.32, df=4, *P*<0.05		*χ*^2^=5.86, df=1, *P*<0.02	*χ*^2^=10.92, *P*<0.05	
Significance of religion		*χ*^2^=1.90 @ df=2 *P*>0.05		*χ*^2^=6.193, df=1, *P*<0.02	*χ*^2^=3.242, df=1, *P*<0.05	
Significance of education of mother		*χ*^2^=24.83, df=6, *P*<0.001		*χ*^2^=24.83, df=6, *P*<0.001	*χ*^2^=10.21, df=3, *P*<0.05	

Table 1 reveal that young mothers significantly had better ambition regarding education of daughters (84%), and economic independence (70%). A total of 63% said no to early pregnancy. As seen in earlier studies(1,2) even we could see Hindus having better ambitions than Muslim mothers. It was evidently seen that educated mothers had better ambition for daughters. Mothers who had completed 10^th^class (55%) had a definite idea what education their daughters should perceive and 86% wanted their daughters to get education higher than 10^th^class. Educated mothers were clearly aware of the early pregnancy risk and wanted their daughters to give birth to child at 20–25 years of age. A total of 80% of the educated mothers wanted their girl child to be economically independent. Mothers with more children had low ambition toward a girl child’s higher education. Socio-economic status was not associated with mother’s ambitions.

Focus group discussion conducted to know indepth understanding of reasons for perception revealed that majority (40%) of the mothers wanted daughters’ economical independence and so also wanted good education; some felt education increases the intelligence and so gives a better life whereas few also felt education is a qualification for marriage. But when the question of not educating daughters arose majority of the mothers had economical constrains (40%) to do so; 30% of the mothers claimed education is of no use as women are not allowed to do a job and housewives do not need education. A total of 20% felt that male child’s education is more important, and so said no to girls’ education.

We found that religion and education of mothers were major factors contributing to better education and health of daughters but SES has no association with mother’s ambitions and perception. Younger mothers were highly ambitious which would move them toward a new era of better women health in near future.

## References

[1] Lal S (1992). Mother’s perception and ambitions about their daughters in rural areas. Indian J Community Med.

[2] Lal S (1997). Mother perceptions and ambitions about their daughters in rural area. Indian J Community Med.

